# Meroterpenoids from Terrestrial and Marine Fungi: Promising Agents for Neurodegenerative Disorders—An Updated Review

**DOI:** 10.3390/cimb47020096

**Published:** 2025-02-03

**Authors:** Daniela Dimitrova, Simeonka Dimitrova, Gabriela Kehayova, Stela Dragomanova

**Affiliations:** 1 Faculty of Pharmacy, Medical University of Varna, Tsar Osvoboditel Blv. 83A, 9000 Varna, Bulgaria; danielladad@icloud.com; 2 Department of Pharmacology, Toxicology and Pharmacotherapy, Faculty of Pharmacy, Medical University of Varna, Tsar Osvoboditel Blv. 83A, 9000 Varna, Bulgaria; simeonka.dimitrova@mu-varna.bg (S.D.); gabriela.kehayova@mu-varna.bg (G.K.)

**Keywords:** neurodegeneration, neuroprotection, anti-cholinesterase, antioxidant, BACE1 inhibition, anti-inflammatory, meroterpenoids, mushroom, fungi, marine sponge

## Abstract

Background: Meroterpenoids represent a remarkably diverse class of natural secondary metabolites, some of which are synthesized via terpenoid biosynthetic pathways. Over the past ten years, these compounds have gained interest because of their wide range of biological activities, such as anti-cholinesterase, COX-2 inhibitory, antibacterial, antiviral, antidiabetic, antioxidant, anti-inflammatory, antineoplastic, and cardioprotective properties. This review aims to consolidate the recognized neuroprotective effects of meroterpenoids from marine and terrestrial fungi. Methods: Data compiled from several databases, including PubMed, Science Direct, Scopus, and Google Scholar, include articles published since 2000 using keywords such as “neuroprotective”, “fungi”, “mushroom”, “marine sponge”, “neurodegeneration”, and “dementia” in connection with “meroterpenoids”. Results: Meroterpenoids modulate different cell signaling pathways and exhibit different and often combined mechanisms of action to ameliorate neuronal damage and dysfunction. Reported activities include anti-cholinesterase, antioxidant, BACE1 inhibition, and anti-inflammatory activities, all of which have potential in the treatment of dementia associated with neurodegenerative diseases such as Alzheimer’s and Parkinson’s. Conclusions: Meroterpenoids have the potential to be developed as effective tools for neuropathological diseases. Ongoing research to elucidate the various neuroprotective pathways remains essential and requires further investigation.

## 1. Introduction

Neurodegenerative diseases (ND) are characterized by the gradual deterioration of neurons, leading to deficits in cognitive abilities, motor functions, and sensory perception. The World Health Organization reports that approximately 55 million people worldwide are affected by dementia, with 60 to 70% of these cases being due to Alzheimer’s disease [1]. Current therapeutic approaches are primarily aimed at alleviating symptoms. However, they fail to alter the long-term course of the disease, a difficulty that also occurs in diseases such as Parkinson’s disease, Huntington’s disease, amyotrophic lateral sclerosis, and other neurodegenerative diseases.

Current approaches to the treatment of neurodegenerative diseases focus on alleviating cognitive and motor symptoms through the use of various pharmacological agents, including cholinesterase inhibitors, dopaminergic drugs, benzothiazoles, and NMDA receptor antagonists [2]. Although these therapies can relieve symptoms, they are often accompanied by a number of side effects. For example, patients with Alzheimer’s disease may experience side effects such as loss of appetite, nausea, diarrhea or vomiting, headache, fatigue, dizziness, and sleep disturbances when treated with donepezil, rivastigmine, and galantamine [3]. Levodopa is initially very effective in Parkinson’s disease, but long-term use can lead to complications such as dyskinesia [4]. In addition, the four approved medications for amyotrophic lateral sclerosis (ALS) and its associated symptoms are linked to various side effects that may include acute chest pain, wheezing, leukopenia, and liver problems [5]. The biggest challenge is finding a balance between relieving symptoms and managing the side effects associated with these medications.

In the search for safer and more effective treatments, meroterpenoids have attracted great interest. Meroterpenoids, derived from the Greek terms méros (partial) and τερέβινθος (terebinth tree), are hybrid compounds composed of terpene units and various other biosynthetic elements. They are a heterogeneous group of natural compounds characterized by both terpene and non-terpene structures. The classification of these compounds lacks a standardized framework, leading to variations due to different criteria and methodologies. These unique molecules originated from a variety of organisms, including plants, fungi, and marine life, and are known for their ability to contact multiple biological targets.

On the one hand, the potential of marine meroterpenoids to influence cell proliferation is known [6]. In 2015, Elissawy et al. directed their research toward biologically active terpenes extracted from marine fungi, specifically examining studies conducted between 2010 and 2014 [7]. Their review highlights 19 newly identified meroterpenoids from this timeframe. On the other hand, the possibilities of these compounds to counteract neurodegenerative processes have not been updated. Two separate 2023 reviews covered the various mushroom-derived metabolites with the potential to influence Alzheimer’s disease [8,9]. But very few of them have considered the potential of meroterpenoids extracted from them to affect neurodegenerative processes.

The increasing importance of meroterpenoids is attributed to their diverse biological activities, especially their neuroprotective effects [10]. Recent research has highlighted the potential of meroterpenoids in counteracting neurodegenerative diseases. The analysis conducted by El-Demerdash et al. (2020) provided a comprehensive overview of the meroterpenoids identified from marine fungi up to that point [11]. The authors categorized the compounds based on their structural characteristics and sources while also enhancing the discussion with the biological properties that have been documented to date. This review material shows a small number of compounds with neuroprotective potential. Out of all 316 molecules included, only nine exhibited anti-cholesterase activity, and two of them showed BACE1-inhibitory activity, which underscores the necessity of revising the data concerning the capacity of this class of compounds to affect neurodegenerative processes. As the majority of meroterpenoids with neuroprotective properties are isolated from plants [12,13,14,15], this review focuses specifically on meroterpenoids derived from fungi. Research shows that the neuroprotective mechanisms of these metabolites include not only the enhancement of neurotransmitters such as acetylcholine and glutamate in the synaptic cleft but also the inhibition of neuroinflammatory pathways, apoptosis, excitotoxicity, mitochondrial damage, and the modulation of BACE1 and BuChE activity and antioxidant and redox modulation [16].

This study represents a comprehensive review of the existing literature, focusing on research conducted since 2000 that highlights the therapeutic potential of terrestrial and marine fungi, particularly their terpene derivatives known as meroterpenoids, in the context of neurodegenerative diseases. A comprehensive search was performed in various databases, including Google Scholar, Web of Science, ScienceDirect, PubMed, Springer Link, and the Virtual Health Library, using a number of keywords and their combinations in English: Alzheimer’s disease, neurodegeneration, neuroprotection, meroterpenoids, fungi, marine sponge, mushroom, amyloid beta, tau, oxidative stress, and enzyme inhibition. In addition, manual searches of reference lists of relevant reports were conducted to identify additional relevant studies. The selection criteria for the relevant studies focused on the application of various meroterpenoid compounds in experimental neurodegeneration models.

## 2. Mechanisms of Neuroprotection

**Figure 1:**
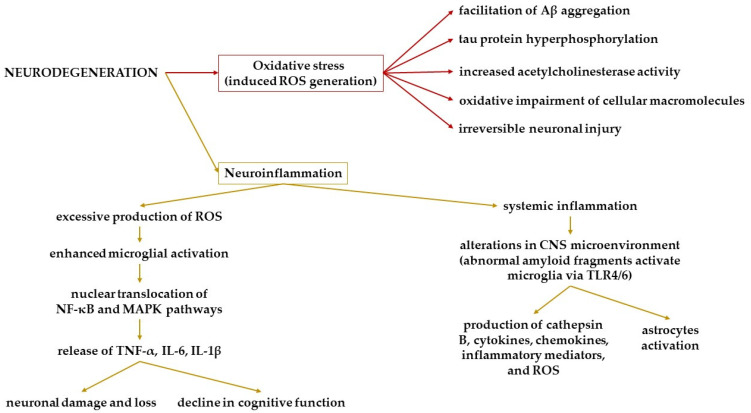
Neurodegeneration mechanisms.

Neuroinflammation and oxidative stress play critical roles in the initiation and advancement of neurodegenerative diseases (Figure 1) [17,18].

On the one hand, the disruption of redox balance results in the buildup of reactive oxygen species (ROS), which subsequently promotes the aggregation of amyloid-beta (Aβ), hyperphosphorylation of tau protein, heightened activity of acetylcholinesterase, and oxidative harm to various cellular macromolecules. This sequence of events ultimately leads to irreversible neuronal injury [19,20]. On the other hand, numerous preclinical and clinical investigations have established a connection between systemic inflammation and alterations in the central nervous system’s microenvironment with neurodegenerative mechanisms [21]. Abnormal amyloid fragments trigger microglial activation through Toll-like receptors 4 and 6 (TLR4/6), resulting in the secretion of cathepsin B, cytokines, chemokines, inflammatory mediators, and ROS while also activating astrocytes. Additionally, the excessive production of reactive free radicals further enhances microglial activation. This process engages various inflammatory signaling pathways, including the nuclear translocation of nuclear factor kappa B (NF-κB) and mitogen-activated protein kinase (MAPK) pathways [17]. The subsequent release of inflammatory mediators such as tumor necrosis factor-alpha (TNF-α), interleukin-6 (IL-6), and interleukin-1β (IL-1β) contributes to neuronal damage, ultimately leading to neuronal loss. This pathological cycle elucidates the deterioration of cognitive functions, including learning and memory [22].

The therapeutic potential of meroterpenoids in neurodegenerative diseases has been better understood as a result of increased research efforts over time, which have also revealed new mechanisms of action. Meroterpenoids terreusterpenes A, B, and D from *Aspergillus terreus* that can inhibit both acetylcholinesterase and BACE1 were first identified in 2018 [23]. In 2019, the same group of researchers explored six novel meroterpenoids from *Aspergillus terreus*, known as spiroterreusnoids A–F [24]. With IC_50_ values for BACE1 and AChE ranging from 5.86 to 27.16 µM and 22.18 to 32.51 µM, respectively, they demonstrated inhibitory activity in vitro. It should be mentioned that this study lacked advanced preclinical evaluations and in vivo models, which opens the door for further research into their possible therapeutic value in neuronal injury models.

**Figure 2:**
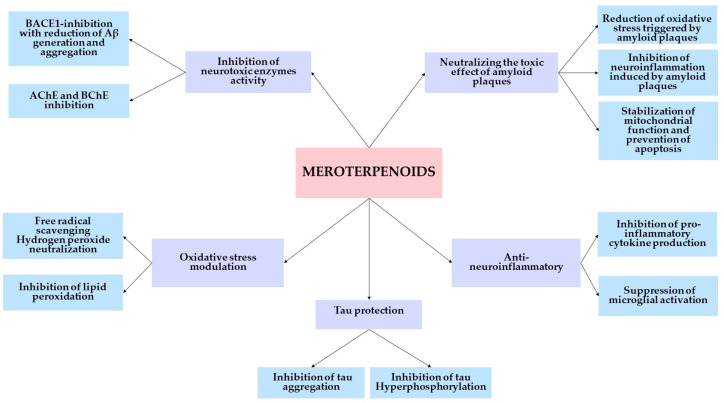
Meroterpenoids’ main mechanisms of neuroprotection.

Numerous studies looking into similar multi-target effects have been prompted by the scientific community’s intense interest in this novel discovery. The most significant neuroprotective mechanisms of action of meroterpenoids that have been discovered thus far are compiled in Figure 2.

### 2.1. Inhibition of Neurotoxic Enzymes

#### 2.1.1. Reducing the Generation and Aggregation of Aβ by Inhibiting BACE1

Aβ plaques are formed primarily from Aβ peptides, which consist of 40 to 42 amino acids. These peptides are formed by the cleavage of the transmembrane protein known as amyloid precursor protein (APP) via the amyloidogenic pathway. This pathway is facilitated by the action of two key enzymes: β-secretase, also called β-site APP-cleaving enzyme 1 (BACE1), and γ-secretase. The initial cleavage of APP is carried out by BACE1, producing a soluble fragment called β-APP and a longer 99 amino acid peptide called C99. Subsequently, γ-secretase acts on the C99 fragment, leading to the formation of Aβ peptides of different lengths, including Aβ-40 and Aβ-42. An imbalance between the production and clearance of Aβ peptides contributes to cell degeneration [25].

Asperterpenes, a class of merotepenoids derived from the soil fungus *Aspergillus terreus*, have been identified as significant inhibitors of BACE1 [26]. To elucidate the BACE1-inhibiting properties of these compounds (particularly asperterpenes A, B, E, F, and G), researchers used a combination of molecular biology techniques, cellular assays and animal models, and in silico target confirmation (ISTC). ISTC testing revealed that asperterpenes A and B have the potential to inhibit BACE1. Notably, asperterpene A had an IC_50_ value of 0.08 μM. In comparison, asperterpene A showed similar activity to LY2811376, the first orally bioavailable non-peptide BACE1 inhibitor, showing an IC_50_ range of 239 nM to 249 nM and an EC_50_ of 300 nM in the context of Alzheimer’s disease at 3xTg-mice. Collectively, these results suggest that asperterpene A represents the first terpenoid found to effectively inhibit BACE1. The IC_50_ values for asperterpenes B, E, F, and J were determined to be 0.06, 3.32, 5.85, and 31.68 μM, respectively. Further, asperterpene E, F, and J derived from *Aspergillus terreus* exhibited notable inhibitory effects on BACE1, with IC_50_ values recorded at 3.3, 5.9, and 31.7 μM, respectively [27].

Terreusterpenes, a group of meroterpenes based on 3,5-dimethylorsellinic acid and also derived from *A. terreus*, showed a significant inhibitory effect against BACE1 [23]. In particular, terreusterpenes A and D showed BACE1 inhibition with IC_50_ values of 5.98 μM and 1.91 μM, respectively. The analysis conducted by El-Demerdash et al. (2020) confirms the ability of terreusterpenes A and B to mitigate the harmful effects of abnormal amyloid plaques through the inhibition of BACE1 [11].

New meroterpenoids, derived from *Boletinus asiaticus*, 14′,15′-dihydroasiaticusin A methyl ester, asiaticusinols A–C, and asiachromenic acid, in addition to the previously identified compound asiaticusin A, exhibited BACE1 inhibitory activity [28]. In a separate investigation, six new and previously identified compounds derived from *Suillus bovinus* and *Boletinus cavipes* were examined [29]. The compounds numbered 1 through 6 exhibited varying degrees of BACE1-inhibitory activity, with IC_50_ values recorded at 21.2, 17.8, 1.0, 1.6, 23.7, and 22.8 μM, respectively.

Table 1 displays meroterpenoids that exhibit inhibitory activity against BACE-1.

#### 2.1.2. Prevention of Acetylcholine Degradation by Inhibiting AChE and BChE

The cognitive decline associated with Alzheimer’s disease is primarily related to the degeneration of cholinergic neurons, which leads to a significant reduction in acetylcholine levels. Cholinesterase inhibitors are pharmacological agents that inhibit the activity of cholinesterases, particularly AChE and BuChE. By preventing the breakdown of the neurotransmitters acetylcholine and butyrylcholine, these inhibitors increase their availability in the central nervous system. Due to its central function in the cholinergic system, AChE has emerged as an important target for therapeutic interventions [9]. Commonly prescribed medications currently include donepezil, rivastigmine, and galantamine. Furthermore, dual cholinesterase inhibitors acting on both AChE and BuChE could allow more balanced cholinergic neurotransmission, potentially leading to improvements in cognitive function and memory. Butyrylcholinesterase, as well as acetylcholinesterase, play a crucial role in the progression of Alzheimer’s disease. One of the main reasons for the resistance of AD to AChE inhibitors is the compensatory function of BuChE in the neurons of Alzheimer’s patients [30]. In addition, deficiency of the neurotransmitter acetylcholine (ACh) has been found to be a major contributor to cognitive decline, with ACh levels predominantly controlled by acetylcholinesterase. Consequently, both BACE1 and the activities of AChE and BChE are considered important therapeutic targets for small molecule inhibitors that have the potential to alter AD progression.

A variety of natural sources have been found to contain compounds with anti-cholinesterase activity, which has attracted great research interest. Among these, three merosesquiterpenes—arisugacin B, arisugacin C, and arisugacin D—were isolated from the endophytic fungus *Penicillium sp. FO-4259* [31]. These compounds showed remarkable inhibitory effects on AChE in vitro, with IC_50_ values recorded at 0.2 nM, 2.5 nM, and 3.5 nM. In addition, arisugacin A, a highly selective AChE inhibitor isolated from *Penicillium sp. FO-4259* showed a high binding affinity with an IC_50_ of 0.001 nM. Arisugacin A was subsequently identified in *Aspergillus terreus*, where it had an IC_50_ of 11.9 nM, in addition to arisugacin D, which had an IC_50_ of 0.39 nM. In a separate investigation, arisugacins D, M, O, P, and Q demonstrated anti-cholinesterase properties [32]. Arisugacin O exhibits the highest inhibitory potency in vitro, with an IC_50_ value of 191 nM; however, its application to zebrafish embryos results in paralysis.

The analysis conducted by El-Demerdash et al. identifies arisugacin B, E, F, and I, along with dehydroaustinol and isoaustinone, as promising candidates for anti-cholinesterase activity in vitro [11]. Additionally, Long et al. (2017) documented that the meroterpenoids isoaustinol, dehydroaustin, and dehydroaustinol exhibit acetylcholinesterase inhibition, with IC50 values recorded at 2.50, 0.40, and 3.0 µM, respectively [33].

Subsequent chemical analysis of the mangrove-derived fungus *Penicillium sp*., obtained from the foliage of the mangrove species *Kandelia candel*, resulted in the identification of several new compounds: 3-epi-arigsugacin E, terreulactone C, territrem B, territrem D, and territrem E [34]. This discovery complements the previously identified arisugacin B and arisugacin D. Notably, the newly identified compounds (arisugacin B, territrem C, and terreulactone C) demonstrated significant inhibitory activity against AChE. The anti-cholinesterase properties of terreulactone A [35] and isoterreulactone A [36] have also been established. Both substances exert their effects in a dose-dependent manner.

Two meroterpenoids, amphichoterpenoids D and E, isolated from *Amphichorda felina SYSU-MS7908*, demonstrate in vitro anti-cholesterase activity, with IC_50_ values of 12.5 μM and 11.6 μM, respectively [37]. Asperversin G from the marine-derived fungus *Aspergillus versicolor* also demonstrated a moderate inhibitory activity against AChE, exhibiting an IC_50_ value of 13.6 μM [38]. Luo et al. (2019) identified five novel meroterpenoids derived from *Ganoderma lucidum* [39]. Notably, compounds dayaolingzhiols D and E demonstrated significant AChE inhibitory effects, with IC_50_ values of 8.52 ± 1.90 μM and 7.37 ± 0.52 μM, respectively.

Other compounds of the large group of meroterpenoids that are of fungal origin and possess anti-cholinesterase activity are as follows: ganocin D from *Ganoderma cochlear* [40]; zizhines M, N, and O from *Ganoderma species* [41]; and ganocapenoid C, ganocalidin E, cochlearin I, and patchiene A from *Ganoderma capense* [42].

Table 2 displays meroterpenoids that exhibit AChE-inhibitory activity.

### 2.2. Oxidative Stress Modulation

Oxidative stress represents a pathological condition characterized by an imbalance between the production of reactive oxygen species and the effectiveness of cellular antioxidant defenses. When ROS levels exceed a critical threshold, it can lead to various deleterious effects, including protein denaturation, lipid peroxidation, induction of apoptosis, and DNA damage. Furthermore, ROS can interact directly with the mitochondrial membrane and disrupt both its structure and function. This disorder triggers a cascade of events that culminate in a decline in neurotransmitter levels, such as acetylcholine and dopamine, which are closely linked to cognitive decline and neuronal death, thereby contributing significantly to the pathogenesis of numerous neurodegenerative diseases [43]. While treating oxidative stress associated with neurodegenerative diseases offers potential therapeutic opportunities, it also presents several challenges. The complex interactions between ROS and various cellular components complicate the identification of specific therapeutic targets due to the intricate nature of the oxidative stress pathways. Furthermore, the heterogeneity of neurodegenerative diseases such as Parkinson’s and Alzheimer’s underlines the need for tailored therapeutic strategies rather than a universal treatment approach. A further complication arises from the fact that, under certain circumstances, antioxidants can exhibit pro-oxidant behavior, reducing their therapeutic efficacy and potentially producing toxic effects [44,45,46].

A recent investigation employed neuronal cell cultures and animal models to explore the various biological characteristics of *Ganoderma lucidum* extract [47]. This study includes an exploration of the compounds’ ability to alleviate oxidative stress by inhibiting the production of ROS and influencing the activity of antioxidant enzymes. Among the over 400 active compounds identified, the meroterpenoids lingzhine E and F exhibited remarkable antioxidant and neuroprotective properties.

Pestalotioquinols A and B, isolated from the plant-associated fungus *Pestalotiopsis microspora*, demonstrated significant neuroprotective properties in neuronal PC12 cells against cytotoxicity induced by peroxynitrite-generated oxidative stress [48].

#### 2.2.1. Free Radical Scavenging

Investigations into *Ganoderma lucidum* have revealed that certain secondary metabolites possess the ability to scavenge free radicals in vitro, thereby providing neuroprotective advantages [49]. The study investigates two aromatic meroterpenoids, lingzhine E and F, which demonstrate the ability to scavenge free radicals in the context of hydrogen peroxide-induced ROS production in SH-SY5Y cells. This capability is supported by results from both 2, 2′-azobis (3-ethylbenzothiazole-6-sulfonic acid) (ABTS) and oxygen radical absorbance capacity (ORAC) assays. Notably, the scavenging activity is associated with a dose-dependent increase in the survival of SH-SY5Y neuroblastoma cells exposed to oxidative stress caused by amyloid-beta, yielding survival rates of 77.11 ± 4.18% and 80.17 ± 5.19%, respectively.

A further study on the meroterpenoids methyl ganoderate G1, lingzhine E, and lingzhine F, which are extracted from *Ganoderma lucidum*, commonly known as the Reishi mushroom, validated their significant ABTS+ (2,2′-azino-bis(3-ethylbenzothiazoline-6-sulfonic acid radical cation) scavenging capabilities [50]. The recorded EC_50_ values were 0.59 ± 0.15 mM and 0.27 ± 0.05 mM, respectively, which are comparable to the positive control, Trolox, which had an EC_50_ of 0.42 ± 0.03 mM. Additionally, the meroterpenoids ganocapensins A and B, along with ganomycin E, F, and I, and fornicin E and B, isolated from *Ganoderma capensa*, exhibited in vitro radical scavenging activity, with IC_50_ values ranging from 6.00 ± 0.11 to 8.20 ± 0.30 μg/mL [51]. The meroterpenoids known as cochlearins A–I, which are extracted from *Ganoderma cochlear*, have demonstrated notable antioxidant properties [52]. Among these, cochlearin D exhibited the most potent radical scavenging activity, with an IC_50_ value of 3.1 ± 0.1 μM, which is comparable to that of the standard antioxidant Trolox. The antioxidant capacity of methyl ganoderate G1, lingzhine E, and lingzhine F was evaluated based on their ability to inhibit oxidation caused by peroxyl radicals. The effectiveness of these natural compounds was compared to the positive control, quercetin. Notably, lingzhine E showed an ORAC value of 7.24 ± 0.27 μmol TE/μmol, which is closely aligned with quercetin’s value of 7.78 ± 0.27 μmol TE/μmol, while lingzhine F recorded an ORAC value of 5.42 ± 0.20 μmol TE/μmol.

Three novel meroterpenoids, perennipin A-C, along with the previously identified fornicin A, were extracted from the wood-decaying fungus *Perenniporia medulla-panis* [53]. These compounds demonstrated antioxidant properties, as evidenced by their IC_50_ values, which varied between 12.8 and 190.3 μM in a radical-scavenging assay. In addition, the secondary metabolites aplanatumols F, aplanatumols H and I, and lingjiol extracted from *Ganoderma sinense* are derivatives of the meroterpenoid fornicin A [54]. These compounds show antioxidant capacity in vitro, with aplanatumol I being the most potent. The hepatoprotective properties of aplanatumol I were demonstrated in an in vitro model using liver cells exposed to H_2_O_2_ damage. This compound effectively protects from H_2_O_2_-induced cell death mediated by caspase-3 by diminishing ROS levels and enhancing the concentration of glutathione and the activity of the antioxidant enzymes superoxide dismutase and catalase. Furthermore, aplanatumol I augmented the cellular antioxidant defense mechanisms through the modulation of the Nrf2 and PI3K/Akt signaling pathways. Additionally, this metabolite exhibited protective effects on cardiomyocytes against ischemic/reperfusion injury. Consequently, the isolated monoterpenoids demonstrate significant potential in addressing various diseases linked to oxidative stress.

#### 2.2.2. Inhibition of Lipid Peroxidation

Lipid peroxidation is defined as the oxidative degradation of lipids, a process initiated by reactive oxygen species that specifically target polyunsaturated fatty acids present in cellular membranes, leading to the formation of lipid peroxides [55].

There is a lack of explicit documentation in the literature concerning specific meroterpenoids that directly inhibit lipid peroxidation. However, the fungi *Ganoderma lucidum* and *Funalia trogii* demonstrate significant inhibitory effects on lipid peroxidation, implying the existence of bioactive compounds, potentially including meroterpenoids [56]. Further research is essential to isolate and identify these meroterpenoids and to assess their antioxidant capabilities.

### 2.3. Tau Protection

Neurofibrillary tangles (NFTs) arise when tau proteins, which typically play a crucial role in maintaining the stability of microtubules in neurons, undergo pathological hyperphosphorylation. This excessive phosphorylation leads to the detachment of tau from microtubules, thereby compromising their structural integrity. Once dislodged, tau proteins begin to misfold, ultimately forming toxic neurofibrillary tangles within the neuronal cell. These tangles interfere with essential cellular processes and contribute to neuronal cell death. Alzheimer’s disease is the most recognized tauopathy [57,58]. In individuals with AD, the presence of misfolded and dysfunctional tau proteins results in the disintegration of the microtubule network. Other tauopathies that have been identified include Huntington’s disease and various other neurodegenerative disorders.

#### 2.3.1. Inhibition of Tau Hyperphosphorylation

A study evaluates the effects of (±)-spiroganoapplanin A and its enantiomers, (+)-1 and (−)-1, on the phosphorylation of tau protein [59]. Western blot analysis demonstrated that (±)-1, (+)-1, and (−)-1 significantly decreased the protein levels of CDK5 while increasing the levels of phospho-GSK3β (pGSK3β) at concentrations of 5 μM and 20 μM. The last indicates that these compounds may inhibit the enzymatic activity of GSK-3β. Furthermore, these meroterpenoids led to a reduction in the levels of phosphorylated tau isoforms, including pTau181, pTau396, and pTau217, which are crucial in the formation of neurofibrillary tangles. The results underscore the potential of (±)-1, (+)-1, and (−)-1 to inhibit tau phosphorylation and its detrimental effects, suggesting their potential as therapeutic agents for Alzheimer’s disease.

#### 2.3.2. Inhibition of Tau Aggregation

- Direct Binding to Tau Protein

Certain meroterpenoids, including austalides, which are derived from *Penicillium species*, engage directly with the tau protein by binding to specific sites that are crucial for the protein’s abnormal aggregation into neurofibrillary tangles [60]. This interaction with designated regions leads to the stabilization of tau in its native, soluble form. Consequently, this mechanism impedes the misfolding of tau and the subsequent development of detrimental oligomers and fibrils.

- Disassembly of Pre-formed Tau Aggregates

Various meroterpenoids, including dictyostatin, obtained from marine sponges, exhibit the ability to interact with pre-formed tau fibrils [59]. This interaction results in the destabilization of the fibrillar structure, promoting disaggregation. Additionally, dictyostatin inhibits the polymerization of tau by binding to the tau protein and stabilizing microtubules, thereby indirectly influencing tau’s tendency to aggregate.

The existing research in this area is still in its preliminary stages and requires further development and expansion.

### 2.4. Anti-Neuroinflammatory Activity

#### 2.4.1. Inhibition of Pro-Inflammatory Cytokine Production

The suppression of pro-inflammatory cytokine synthesis is crucial for maintaining neuronal integrity and safeguarding cerebral cells from potential harm. Excessive production of cytokines, including TNF-α, IL-1β, and IL-6, has been associated with the pathophysiological processes of neuroinflammation. This imbalance may result in the disruption of the blood–brain barrier, the onset of oxidative stress, and the activation of microglial cells, all of which can exacerbate a range of neurological conditions. The inflammatory cascade involved ultimately results in neuronal injury, synaptic impairment, and cell death, which are key mechanisms that contribute to neurodegenerative diseases.

Meroterpenoids exhibiting anti-inflammatory characteristics have been extracted from marine sponges belonging to the *Dysideidae* family. Specifically, dysivillosins A–D were identified in *Dysidea villosa* [61]. Dysivillosin A suppresses the production of the pro-inflammatory cytokine interleukin-4 (IL-4) in mast cells.

Aspertetranones A-D represent a class of highly oxygenated triketide-sesquiterpenoid meroterpenes, which have been isolated from a fungus associated with marine algae, specifically *Aspergillus* sp. [62]. The anti-inflammatory effects of these compounds were examined in RAW264.7 macrophages stimulated with lipopolysaccharide (LPS). Aspertetranones A and D demonstrated a dose-dependent inhibition of IL-6 and IL-1β production among the substances studied. Dysiarenone, a meroterpenoid derived from the marine sponge *Dysidea arenaria*, has been shown to inhibit the expression of COX-2 and the production of PGE2 [63].

Another 2024 study on andrastin-type meroterpenoids from the marine fungus *Penicillium chrysogenum* highlights the remarkable anti-neuroinflammatory properties of newly discovered compounds, particularly penimerodione A [64,65,66]. This compound showed significant inhibition of nitric oxide (NO) production in LPS-stimulated BV-2 microglial cells, indicating its potential to alleviate neuroinflammation, a crucial element in the progression of neurodegenerative diseases. Penimerodione A potently inhibited inflammatory proteins such as iNOS and COX-2 at an IC_50_ of 5.9 μM by targeting the MAPK signaling pathway. This effect establishes andrastine-type meroterpenoids as potential agents to alleviate neuroinflammatory diseases and strengthens the viability of marine fungi as sources of bioactive compounds to counteract inflammation-induced neurodegeneration.

In two separate studies conducted in 2018, Luo et al. demonstrated that meroterpenoids ganotheaecolumols C, D, I, K, and iso-ganotheaecolumol I derived from *Ganoderma theaecolum* possess the capability to inhibit COX-2 activity with IC_50_ values varied between 1.05 and 4.84 μM, and ganotheaecoloid J with IC_50_ value of 9.96 μM [67]. Applanatumol C, derived from *Ganoderna applanatum*, inhibits COX-2 with an IC_50_ value of 25.5 μM [68].

Meroterpenoids extracted from *Ganoderma cochlear*, spirocochlealactones A-C, and ganodilactone exhibit the capability to inhibit COX-2, with IC_50_ values ranging from 1.29 to 3.63 μM [69], as well as gancochlearols A and B [70]. Furthermore, cochlactones A and B, also derived from *Ganoderma cochlear*, exhibited anti-inflammatory properties by effectively inhibiting NO production, with IC_50_ values of 5.9 ± 0.1, 6.1 ± 0.2, 18.7 ± 1.9, and 12.1 ± 0.4 μM, respectively, which were more potent than the positive control [71].

Ganoresinoid A, a meroterpenoid derived from the edible Ganoderma resinaceum, demonstrates the ability to inhibit the production of NO, interleukin-1 beta (IL-1β), interleukin-6 (IL-6), and tumor necrosis factor-alpha (TNF-α) in vitro within LPS-stimulated microglial cells [72]. This inhibition occurs through the suppression of the NF-κB and MAPK signaling pathways. Ganoresinoid A exhibits additional properties that may affect neurodegenerative processes, including the reduction of LPS-induced apoptosis by lowering mitochondrial membrane potential and ROS levels. Furthermore, it displays antioxidant effects in vitro in SH-SY5Y cells subjected to H_2_O_2_-induced oxidative stress by activating the Akt/GSK-3β/Nrf2 signaling pathway.

Meroterpenoids chrysogenolide C, D, and F; berkeleyacetal C; and purpurogenolide C, which were isolated from the endophytic fungus *Penicillium chrysogenum*, demonstrated inhibitory effects on NO production, with IC_50_ values recorded at 78.2 ± 0.3, 24.0 ± 1.7, 12.7 ± 0.6, 4.3 ± 1.2, and 20.9 ± 1.4 μM, respectively [65]. In a separate investigation, purpurogenolides B, C, and D, along with berkeleyacetal C, which have been isolated from the fungus *Penicillium purpurogenum*, showed anti-inflammatory properties [73]. This activity is attributed to a similar mechanism of action in lipopolysaccharide-activated BV-2 microglial cells, with IC_50_ values ranging from 0.8 to 30.0 μM. Additionally, stachybotrysin C, stachybonoid F, and stachybotrylactone, derived from the crinoid-associated fungus *Stachybotrys chartarum*, exhibit moderate anti-inflammatory potential [74]. This is evidenced by their ability to inhibit NO production in LPS-activated RAW264.7 cells, with IC_50_ values recorded at 27.2, 52.5, and 17.9 mM, respectively. Zhang et al. (2018a) utilized the same in vitro model and discovered that brasilianoid B and C from the sponge-associated fungus *Penicillium brasilianum* exhibited moderate anti-inflammatory effects [75].

Mangiterpene C, derived from *Guignardia mangiferae*, exhibited an inhibitory effect on the LPS-induced production of NO, with an IC_50_ value of 5.97 μM [76]. Furthermore, its anti-inflammatory properties were evidenced by the suppression of the NF-κB signaling pathway and a reduction in the expression levels of various inflammatory mediators.

Research has independently examined the anti-inflammatory properties of meroterpenoids derived from *Aspergillus terreus*. In 2015, Liaw and colleagues identified Yaminterritrem B as a COX-2 inhibitor [77], while in 2018, Liu et al. referenced 1,2-dehydroterredehydroaustin as a moderate inhibitor of NO production [78] as the key compounds contributing to the anti-inflammatory effects.

Amestolkolide B, derived from the mangrove endophytic fungus *Talaromyces amestolkiae*, demonstrated significant anti-inflammatory properties in vitro [79]. The meroterpenoid effectively reduced NO production in LPS-activated RAW264.7 cells, exhibiting an IC_50_ value of 1.6 ± 0.1 μM.

The neuroprotective potential of *Hericium erinaceus* has been found in a variety of conditions, including animal models of Alzheimer’s [80] and Parkinson’s disease [81], age-related memory impairment, and more. Administering a standardized extract to Alzheimer’s patients improves memory compared to a placebo-controlled group, according to a 2020 study [82]. It is believed that the main active principles in this mushroom are meroterpenoids erinacins. The in vitro neuroprotective effect of erinacin A is primarily attributed to its anti-neuroinflammatory and anti-apoptotic properties [83], as well as its ability to stimulate the synthesis of nerve growth factor [84]. Furthermore, the substance demonstrated beneficial properties in experimental Parkinson’s disease by maintaining the viability of dopaminergic neurons both in vitro and in vivo and preventing neuroinflammation triggered by the activation of microglia [85]. Additionally, this meroterpenoid enhances the synthesis of NGF and catecholamines in the locus ceruleus and hippocampus of experimental rats, whereas it inhibits their production in the cortex [86], while in mice, a decrease in the number of apoptotic neurons was observed [80].

#### 2.4.2. Suppression of Microglial Activation

In neurodegenerative diseases, inflammation acts as a protective mechanism against infections, trauma, aging, and dementia, among other detrimental elements. The central nervous system’s neuroimmune cells, known as microglia, are at the core of this response. Microglia typically show little immunoreactivity, but they become active in response to stimulation or injury. There are two types of activated microglia: pro-inflammatory M1, which can injure, and anti-inflammatory M2, which promotes regeneration and offers neuroprotection [87]. Atypical phagocytosis, which primarily affects healthy neurons and contributes to neurodegenerative diseases, can result from prolonged microglial activation. It is crucial to create medications that target these processes in order to treat NDDs, as altering microglial activation has demonstrated promise in reducing neuroinflammation and neuronal degeneration [88]. Microglia can be activated by NF-κB following neurological damage, leading to the release of pro-inflammatory substances such as TNFα, IL-1β, and reactive oxygen species [89]. This activation further aggravates neurodegenerative mechanisms and plays a role in secondary neurotoxicity. Inhibition of NF-κB activity has been demonstrated to diminish the activation of M1 microglia, consequently reducing neuroinflammatory responses.

An investigation identified the meroterpenoid dysiherbol A, derived from the marine sponge *Dysidea arenaria*, as a strong inhibitor of NF-κB, exhibiting IC_50_ values of 0.49 [90]. This suggests its potential efficacy in mitigating neuroinflammation. Septosone A, a compound classified as a meroterpenoid and sourced from the marine sponge *Dysidea septosa*, demonstrates a similar mechanism of action. It has been shown to inactivate the NF-κB signaling pathway in CuSO_4_-induced transgenic fluorescent zebrafish [91].

Fifteen meroterpenoids have been isolated from the sponge-associated fungus *Alternaria* sp., with thirteen of these compounds displaying anti-inflammatory activity [92]. These substances—namely tricycloalternarenes A, B, and C; bicycloalternarenes A, B, C, D, and F; and monocycloalternarenes A, B, Cm, and D—are capable of inhibiting the NF-κB signaling pathway, indicating their potential to mitigate neuroinflammation linked to the activation of microglia.

The exploration of neuroinflammation within the context of neurodegenerative diseases has revealed the promising potential of *Ganoderma lucidum* in attenuating microglial activation, as evidenced by numerous studies. In a pivotal study conducted in 2011, Zhang et al. demonstrated that extracts of *Ganoderma lucidum* (GLE) significantly inhibited microglial activation by diminishing the synthesis of pro-inflammatory cytokines, including TNF-α and IL-1β, alongside reactive oxygen species such as nitric oxide and superoxide [93]. These inhibitory effects were found to be dose-dependent. In co-cultures of microglia and dopaminergic neurons, *G. lucidum* exhibited protective effects against neurotoxicity induced by lipopolysaccharide or MPP+, primarily by maintaining dopamine uptake through the suppression of NF-κB activation, which is a key driver of inflammatory and pro-apoptotic pathways. These observations underscore the significance of *G. lucidum* in the modulation of inflammatory processes associated with neurodegenerative disorders. While the initial findings affirm the capacity of *Ganoderma lucidum* to inhibit microglial activation and alleviate neuroinflammation, subsequent investigations aim to elucidate the specific compounds and signaling mechanisms involved. Additional research indicates that GLE also reduces the expression of critical inflammatory cytokines, such as MIP3α, RANTES, G-CSF, IL1α, and MCP-5, with MIP3α being nearly completely suppressed [94]. Furthermore, GLE has been shown to inhibit the expression of genes such as CHUK, NFκB1/p50, and IKBKE, which are essential regulators of the NFκB and MAPK signaling pathways. An important direction for future research could be the identification and characterization of the active compounds within this mushroom extract that contribute to its anti-inflammatory effects.

### 2.5. Neutralization of Amyloid Plaques Toxicity

The pathological cascade that results in amyloid plaque toxicity begins with the misfolding and oligomerization of Aβ peptides. These oligomers disrupt intracellular signaling and cell membranes, which leads to the formation of plaques extracellularly. These plaques change the neuronal microenvironment, attract immune cells, and cause inflammation [95]. By inhibiting Aβ peptides from initially aggregating, meroterpenoids may reduce plaque burden and subsequent toxicity.

A sesterterpene meroterpenoid identified in the mushroom *Hericium erinaceus*, known as erinacine S, demonstrated a reduction in the toxicity associated with Aβ plaques following a 30-day oral administration in APP/PS1 transgenic mice [96].

Four novel meroterpenoids, specifically scutigeric acid, albatrelactone methyl ester, albatrelactone, and 10′,11′-dihydroxygrifolic acid, were isolated from *Albatrellus yasudae* [97]. These compounds, along with the methyl ester of scutigeric acid, demonstrated inhibitory effects on Aβ aggregation and also exhibited inhibitory activity against beta-site APP-cleaving enzyme (BACE1). A recent investigation examined the inhibitory effects of ten meroterpenoids derived from the same mushroom, *Albatrellus yasudae*, on amyloid beta aggregation [98]. Among these, three compounds were newly characterized: 2-hydroxy-1-methoxy neogrifolin (1), 9′-keto-grifolic acid (2), and bis-2-hydroxy-1-methoxy neogrifolin (3). Additionally, seven previously identified meroterpenoids were included: grifolin (4), grifolic acid (5), neogrifolin (6), confluentin (7), 2-hydroxyneogrifolin (8), daurichromenic acid (9), and a cerebroside derivative (10). Notably, compounds 1, 3, 5, 6, 8, and 9 exhibited potential as inhibitors of amyloid beta aggregation, with bis-2-hydroxy-1-methoxy neogrifolin demonstrating IC_50_ values that were closest to those of the reference compound myricetin.

#### 2.5.1. Reduction of Oxidative Stress Triggered by Amyloid Plaques

Oxidative stress and Aβ plaque formation are linked components of Alzheimer’s disease that reinforce one another in a destructive neurodegenerative cycle. By interacting with metal ions and compromising mitochondrial function, Aβ peptides aggregate into plaques that release ROS [99]. By activating the enzymes that produce Aβ, oxidative stress damages and compromises the health of neurons, cellular lipids, proteins, and DNA and promotes further Aβ synthesis [100]. This cycle increases the pathology of Alzheimer’s disease and speeds up cognitive decline by exacerbating inflammation, mitochondrial impairment, and synaptic damage.

Research on substances from *Ganoderma lucidum* that reduce oxidative stress brought on by amyloid-β plaques, a major contributor to the pathophysiology of Alzheimer’s disease, is described in a study [50]. Among the isolated compounds, methyl ganoderate G, lingzin E, and lingzin F demonstrated notable antioxidant and neuroprotective effects. The compounds reduced the cytotoxic effects of Aβ in experiments using SH-SY5Y neuroblastoma cells, which are known to be susceptible to oxidative stress. Aβ25–35, a frequently researched Aβ fragment, decreased cell viability to 63–43% of control values. Cell survival rates rose to 72.4%, 77.11%, and 80.17%, respectively, after treatment with 40 μM of compounds 1, 6, and 7, suggesting dose-dependent protection against Aβ-induced damage. Using a DCFH-DA probe, these substances dramatically decreased intracellular ROS levels, a sign of oxidative stress, compared to untreated cells. With an ORAC value of 7.24 µmol Trolox equivalents/µmol, Lingzhine F (7) demonstrated the highest antioxidant capacity and was close to the quercetin benchmark of 7.78 µmol. ABTS tests for radical scavenging activity revealed that Lingzhin F significantly neutralized reactive oxygen species, with an EC_50_ of 0–27 mM compared to 0–42 mM for the standard antioxidant Trolox. By directly lowering ROS levels and shielding cells from neurotoxicity, compounds derived from *Ganoderma lucidum* may function as natural antioxidants and mitigate Aβ-associated oxidative damage in Alzheimer’s disease, according to the findings.

#### 2.5.2. Inhibition of Neuroinflammation Induced by Amyloid Plaques

Alzheimer’s disease pathology is closely associated with neuroinflammation, an immune response marked by glial cell activation and the release of inflammatory mediators. Pro-inflammatory cytokines are essential for the development of AD from mild cognitive impairment. The brain’s intrinsic immune cells, known as microglia, play a key role in this process. They release inflammatory cytokines, chemokines, reactive oxygen species, nitric oxide, and other tumor necrosis factor-alpha (TNF-α) upon Aβ binding to their receptors.

In the aforementioned study by Peng et al. (2022), the meroterpenoid (±)-spiroganoapplanin A isolated from *Ganoderma applanatum*, through BACE1-inhibition, in addition to suppressing tau-hyperphosphorylation, also suppresses amyloid plaque formation [59]. The compound and its isomers influence the CDK5 and GSK3β signaling pathways, which are two kinases that play a critical role in the formation of amyloid plaques and the hyperphosphorylation of tau, both of which are integral to the neurodegenerative process. Consequently, these compounds function as multi-target agents, possessing the potential to impact neurodegenerative processes through various mechanisms.

#### 2.5.3. Stabilization of Mitochondrial Function and Prevention of Apoptosis

Maintaining mitochondrial membrane potential (Ψm) is crucial for cell division, ROS generation, and oxygen sensitivity. Cell death often results from a decrease in mitochondrial membrane potential, which triggers mechanisms to eliminate dysfunctional mitochondria. Mitochondrial health reduces neuronal mortality and stabilizes neuronal networks and cognition.

Meroterpenoids maintain cellular energy and block apoptosis pathways caused by amyloid plaques. Applanatumol I effectively preserved the mitochondrial membrane potential (MMP) in LO2 liver cells during H_2_O_2_-induced oxidative stress [101]. Exposure of the cells to 250 μM H_2_O_2_ for 6 h resulted in loss of MMP and reduced cell viability. However, 200 μM of the meroterpenoid before treatment significantly prevented MMP decrease, indicating its protective effect on mitochondrial function during oxidative stress. Fluorescence microscopy showed a significant decrease in intracellular levels of reactive oxygen species. H_2_O_2_ exposure decreased the activity of antioxidant enzymes such as SOD, CAT, and GSH, but their activity was restored [102].

The flavonoid-triterpene-saponin meroterpenoids clinoposides G and H, which were isolated from *Clinopodium chinense*, demonstrated protective properties against anoxia/reoxygenation (A/R)-induced apoptosis in H9c2 cells [103]. This is because they promote the normally impaired ΔΨm, which is maintained during A/R stress, causing mitochondrial depolarization and apoptosis. These substances decrease pro-inflammatory cytokines like TNF-α, IL-6, IFN-γ, and MCP-1 while increasing the activity of antioxidant enzymes like superoxide dismutase and catalase. By increasing Nrf2 expression, clinoposides G and H mechanistically boost the synthesis of antioxidant proteins and inhibit NF-κB-p65, a master regulator of inflammation and apoptosis. These substances have potential as treatments for oxidative stress-induced myocardial damage because they inhibit mitochondrial dysfunction and apoptosis by controlling inflammation and oxidative stress.

The focus on cardiomyocytes and hepatocytes implies that the mechanisms that have been identified—namely, Nrf2 activation, improved antioxidant defense, and stabilization of mitochondrial function—point to potential neuroprotective advantages. Applanatumol I and clinoposisides G and H can have protective effects through similar mechanisms in neurodegenerative diseases marked by oxidative stress, mitochondrial dysfunction, chronic inflammation, and apoptosis [99,100]. Their potential to slow the progression of neurodegenerative diseases by modifying oxidative and inflammatory processes in neurons is demonstrated by their ability to inhibit NF-κB signaling, which may also reduce neuroinflammation.

Endoplasmic reticulum (ER) stress impairs the protein folding mechanisms, resulting in the accumulation of misfolded proteins within the ER. This buildup ultimately interferes with cellular functions, contributing to the progression and development of neurodegenerative conditions. The meroterpenoid 3-hydroxyhericenone F, derived from the mushroom *Hericium erinaceum*, demonstrated a protective effect against cell death in Neuro2a cells induced by endoplasmic reticulum stress [104].

Sixteen secondary metabolites, identified as triterpenoids and meroterpenoids, have been isolated from the medicinal mushroom *Ganoderma leucocontextum*, demonstrating neuroprotective properties [105]. Ganoleucoins Q and R demonstrated combined in vitro protective effects via enhancing the viability of PC12 cells against H_2_O_2_-induced damage and facilitating neurite outgrowth.

### 2.6. Other Neuroprotective Mechanisms of Meroterpenoids

Neurotrophins, such as nerve growth factor (NGF) and brain-derived neurotrophic factor (BDNF), play an essential role in the central nervous system function. Numerous terpenoids derived from mushrooms have demonstrated the ability to enhance neurite outgrowth. Hericenones are a group of meroterpenoids isolated from *Hericium erinaceum* (Lion’s mane mushroom). Together with other terpenes derived from this source, they have shown the ability to stimulate NGF synthesis in mouse astroglial cells [106]. Hericenones F, G, and H were found to have the highest potential to affect neuronal growth in the indicated manner [107]. Dictyophorines A and B, derived from the mushroom *Dictyophora indusiata*, have been shown to enhance the synthesis of NGF in astroglial cells [108]. Corallocins A-C, derived from the mushroom *Hericium coralloides*, have been shown to stimulate the expression of NGF and/or BDNF in human 1321N1 astrocytes [109].

Yan et al. (2015) successfully extracted 12 meroterpenoids from the fungus *Ganoderma lingzhi* [110]. Among these compounds, spirolingzhine A exhibited the most significant enhancement of neural stem cell proliferation as a manifestation of its neuroprotective potential.

Eleven meroterpenoids, bistachybotrysins L-V, were extracted from the fungus *Stachybotrys chartarum* [111]. Among these, bistachybotrysins M, N, and T demonstrated a neuroprotective effect against glutamate-induced toxicity in vitro, enhancing cell viability. Additionally, bistachybotrysin S exhibited anti-inflammatory properties by inhibiting LPS-induced nitric oxide production in BV2 cells.

*Poria cocos* (*Polyporaceae* family) is a saprophytic fungus characterized by its abundant triterpenoid content [112]. The essential oil derived from this mushroom exhibits in vivo neuroprotective characteristics, which are expressed through a variety of different mechanisms [113]. A notable enhancement in memory functions has been observed, correlated with a reduction in acetylcholinesterase activity in experimental rats with neurodegeneration induced by Aβ1-40. The beneficial effects also include heightened activities of the antioxidant enzymes catalase (CAT), glutathione peroxidase (GPx), glutathione S-transferase (GST), and glutathione reductase (GR). Unfortunately, there is no data on which of the active principles identified in the mushroom are responsible for the established neuroprotective properties.

Ganomycin C, ganoresinain A, and ganotheaecoloid G derived from *Ganoderma australe* have been shown to mitigate neural excitotoxicity in SH-SY5Y cells that is triggered by glutamate [114]. Furthermore, fischerin, derived from the *Neosartorya fischeri* strain JS0553 isolated from the leaves of *Glehnia littoralis*, demonstrated a neuroprotective effect against glutamate-induced cytotoxicity in HT22 cell lines [115].

Cochlearoids A and C, along with cochlearine A, which are extracted from *Ganoderma cochlear*, exhibit a substantial inhibitory effect on T-type calcium channels [116]. This characteristic suggests their considerable potential in the therapeutic management of various neurological disorders.

The identified *Ganoderma applanatum* compound (±)-ganoapplanin A is categorized within a new class of Ganoderma meroterpenoid dimers. Ganoapplanin, identified as an inhibitor of T-type voltage-gated calcium channels with an IC_50_ value of 36.6 μM, represents a promising candidate for the advancement of therapeutic strategies aimed at neurodegenerative disorders [101].

## 3. Future Directions

The review highlights the substantial potential of meroterpenoids derived from both terrestrial and marine fungi in the treatment of neurodegenerative diseases. However, it also identifies significant gaps in the existing literature that warrant further investigation. The current system for classifying meroterpenoids lacks standardization, leading to inconsistencies in research outcomes. It is crucial for future research efforts to establish a unified classification framework that facilitates effective comparisons across various studies and enhances the understanding of the biological roles of these compounds.

Moreover, while the review identifies a limited number of meroterpenoids with neuroprotective properties, the majority of investigations have predominantly focused on plant-derived compounds. There is an urgent need for additional studies to examine a broader spectrum of meroterpenoids originating from fungi and to clarify their specific neuroprotective mechanisms. Furthermore, many of the compounds identified thus far have not undergone sufficient preclinical and clinical evaluations. Future research should prioritize in vivo studies to assess the efficacy and safety of meroterpenoids in neurodegeneration models, along with their pharmacokinetic profiles and potential side effects.

Although various mechanisms of action have been referenced, a more in-depth understanding of the molecular pathways influenced by meroterpenoids is essential. Research should aim to elucidate the specific signaling pathways that mediate their neuroprotective effects, particularly with respect to neuroinflammation, oxidative stress, and mitochondrial function. Additionally, the potential for synergistic interactions among meroterpenoids or their combinations with existing therapeutic agents remains largely unexplored. Future studies should investigate the combined effects of meroterpenoids and other pharmacological treatments to enhance therapeutic efficacy, as well as to enhance the understanding of representatives possessing multiple mechanisms of action related to neurodegenerative processes.

## 4. Conclusions

Meroterpenoids exhibit a range of neuroprotective properties, including antioxidant, anti-inflammatory, mitochondrial stabilization, anti-cholinesterase activity, and the inhibition of misfolded protein formation. These characteristics position them as a promising category of compounds for mitigating the advancement of neurodegenerative disorders. The review primarily concentrates on specific fungal species; thus, expanding the scope to encompass a wider range of fungal taxa may uncover novel meroterpenoids with unique neuroprotective properties. By addressing these research gaps, future investigations have the potential to significantly augment the therapeutic capabilities of meroterpenoids. Ongoing research into their multiple mechanisms of action and therapeutic efficacy is essential for optimizing these compounds as effective treatments for neurodegenerative diseases.

## Figures and Tables

**Table 1:** Meroterpenoids with BACE-1 inhibitory activity.

Name	Value, µM	Ref.
Asperterpene B	0.06	[26]
Asperterpene A	0.08	[26]
Compound 3 (*Suillus bovinus*)	1.0	[29]
Compound 4 (*Boletinus cavipes*)	1.6	[29]
Terreusterpene D	1.91	[23]
Asiaticusin A	2.00	[28]
Asperterpene E	3.3	[27]
Spiroterreusnoid A	5.86	[24]
Asperterpene F	5.9	[27]
Terreusterpene A	5.98	[23]
Asiachromenic acid	11.4	[28]
Terreusterpene B	11.42	[23]
Asiaticusinol C	14.7	[28]
Compound 2 (*Suillus bovinus*)	17.8	[29]
Compound 1 (*Suillus bovinus*)	21.2	[29]
Spiroterreusnoid C	21.34	[24]
Compound 6 (*Boletinus cavipes*)	22.8	[29]
Compound 5 (*Boletinus cavipes*)	23.7	[29]
Spiroterreusnoid D	24.98	[24]
Spiroterreusnoid F	25.36	[24]
Spiroterreusnoid B	25.55	[24]
Spiroterreusnoid E	27.16	[24]
Asperterpene J	31.7	[27]

**Table 2:** Meroterpenoids with AChE-inhibitory activity.

Name	Value, nM	Ref.
Arisugacin A	1.0	[31]
Cyclophostin	1.3	[31]
Territrem C	6.8	[31]
Territrem B	7.6	[31]
Arisugacin B	25.8	[31]
**Name**	**Value, µM**	**Ref.**
Terreulactone C	0.028	[34]
Terreulactone A	0.2	[35]
Territrem C	0.23	[34]
Dehydroaustin	0.4	[33]
Isoaustinol	2.5	[33]
Isoterreulactone A	2.5	[36]
Dehydroaustinol	3.0	[33]
Arisugacin B	3.03	[34]
Dayaolingzhiol E	7.32	[39]
Cochlearin I	8.2	[42]
Dayaolingzhiol D	8.52	[39]
Terreusterpene D	8.86	[23]
Amphichoterpenoid E	11.6	[37]
Amphichoterpenoid D	12.5	[37]
Zizhine O	12.67	[41]
Zizhine M	13.19	[41]
Asperversin G	13.6	[38]
Ganocalidin E	18.7	[42]
Spiroterreusnoid A	22.18	[24]
Spiroterreusnoid C	23.87	[24]
Zizhine N	24.78	[41]
Patchiene A	26.0	[42]
Spiroterreusnoid D	26.85	[24]
Spiroterreusnoid B	27.36	[24]
Ganocapenoid C	28.6	[42]
Spiroterreusnoid F	31.33	[24]
Spiroterreusnoid E	32.51	[24]

## Data Availability

Not applicable.
